# Treatment of pediatric central nervous system infection caused by carbapenem-resistant *Klebsiella pneumoniae* with ceftazidime/avibactam, two cases report and literature review

**DOI:** 10.3389/fphar.2025.1532608

**Published:** 2025-02-21

**Authors:** Lu Qing, Haiyang Zhang, Xin Pan, Zhongqiang Liu

**Affiliations:** ^1^ Department of Pediatrics, West China Second University Hospital, Sichuan University, Chengdu, Sichuan, China; ^2^ Key Laboratory of Birth Defects and Related Disease of Women and Children (Sichuan University), Ministry of Education, Chengdu, Sichuan, China; ^3^ Department of Pediatric Intensive Care Unit, West China Second University Hospital, Sichuan University, Chengdu, Sichuan, China

**Keywords:** ceftazidime/avibactam, carbapenem-resistant *Klebsiella pneumoniae*, central nervous system infection, pediatric intracranial infection, literature review

## Introduction

Bacterial resistance has become an increasingly serious problem in recent years, leading to a rapid rise in infections caused by carbapenem-resistant *Klebsiella* pneumonia (CRKP) (Mohamed et al., 2024; Shi et al., 2024). First reported in 1986 (Liu et al., 1986), hypervirulent KP (hvKP) can cause liver abscess, meningitis, pulmonary embolism. It exhibits enhanced pathogenicity and transmissibility, posing greater challenges to clinical treatment, especially with carbapenem resistant (CR-hvKP). One of the most severe complications of CR-hvKP infection is meningitis, which is associated with high mortality and morbidity, underscoring the urgent need for effective therapeutic strategies.

Currently, the treatment options for CR-hvKP meningitis are limited, and their efficacy remains suboptimal. Polymyxin B has been reintroduced for treating carbapenem-resistant Gram-negative bacteria (CRGNB) through intrathecal administration, showing some efficacy in cases of CRGNB meningitis (Ye et al., 2022). However, resistance to polymyxin B has emerged in some CRGNB strains, complicating the management of CRKP-related intracranial infections and further increasing the risks of mortality and disability. Ceftazidime/avibactam (CAZ/AVI), a novel combination of a β-lactam antibiotic and a β-lactamase inhibitor, has demonstrated promising results in treating multidrug-resistant bacterial infections. While CAZ/AVI is primarily approved for treating intra-abdominal infections, urinary tract infections, and hospital-acquired/Ventilator-Associated pneumonia, there are few reports on its use for treating CR-hvKP meningitis in children. Therefore, further investigation is needed to assess its effectiveness in central nervous system infections.

This study reported two cases of CRKP meningitis treated with CAZ/AVI, evaluating its efficacy and safety and providing valuable reference information for clinicians. Additionally, we have summarized and analyzed previously reported cases to offer a clearer understanding of the status of CAZ/AVI in treating central nervous system infections caused by CRKP in children, thereby laying a foundation for future research.

## Case presentation

### Case 1

**FIGURE 1:**
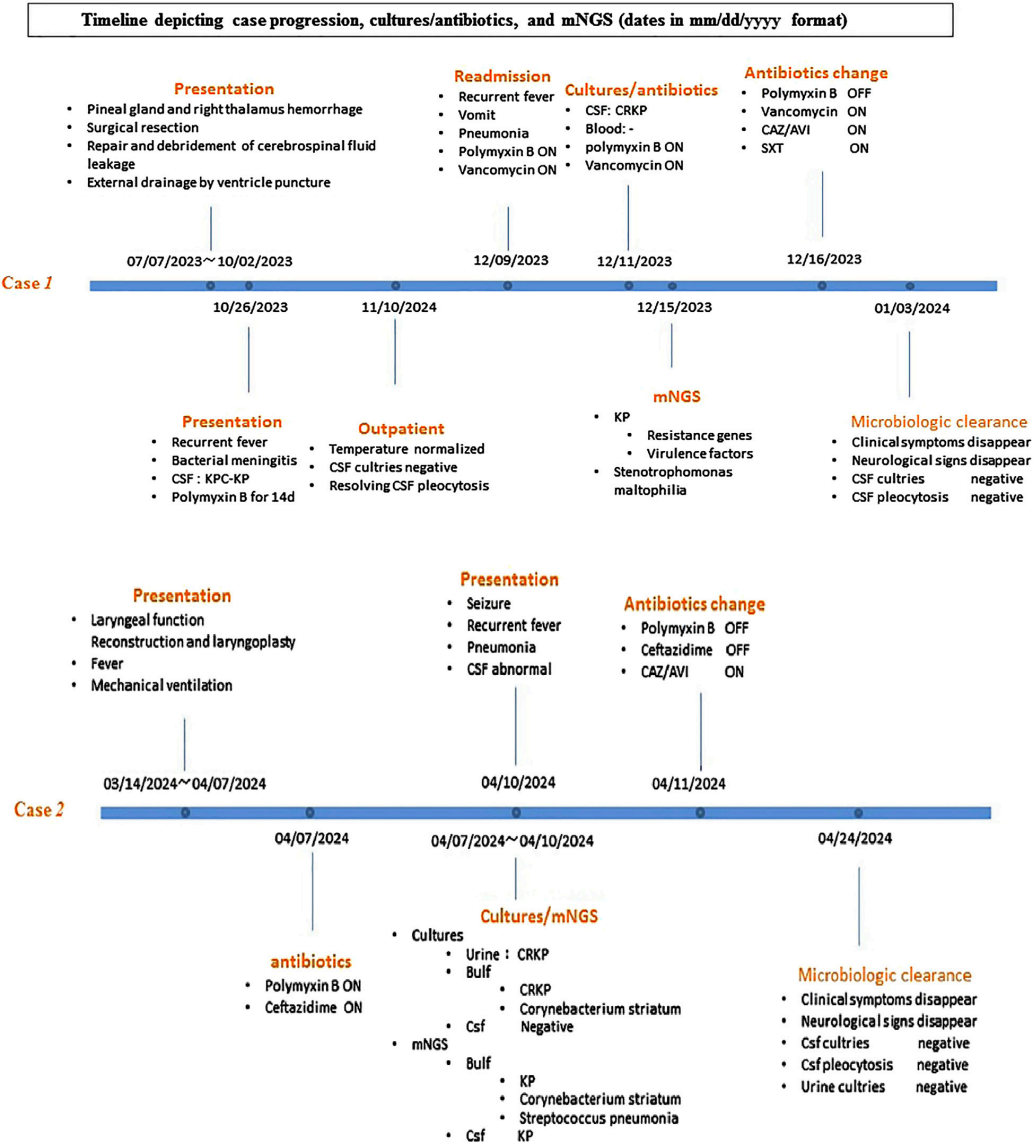
Timeline depicting case progression, cultures/antibiotics, and mNGS (dates in mm/dd/yyyy format).

In December 2023, a 7-month-old male infant was admitted to the Pediatric Intensive Care Unit of West China Second University Hospital due to recurrent fever with vomit (see Figure 1). Four months earlier, the child had developed an intracranial infection after brain surgery. CSF culture confirmed CRKP infection. After 14 days course of polymyxin B (50,000 U/kg/day, intravenous drip every 12 h, 30,000 U, daily intrathecal injection), the CSF findings normalized, and the patient was discharged after recovery.

One week before admission, the patient experienced recurrent fever, peaking with 39.1°C and vomiting 7-8 times daily. The external intraventricular drainage tube was firmly fixed in the abdomen, with cloudy CSF observed in drainage tube. CSF samples obtained from lumbar puncture and drainage tube showed CSF pleocytosis (white blood cell count [WBC] 967/mm^3^, 55% polymorphonuclear cells), low CSF glucose (2.32 mmol/L), and high CSF protein (352.4 mg/dL). CSF culture and antimicrobial susceptibility testing confirmed the growth of KPC-producing *Klebsiella pneumoniae* (KPC-KP), which was sensitive to tigecycline and resistant to polymyxin B, cefoperazone-sulbactam, meropenem, imipenem and levofloxacin. The E-test with CAZ/AVI result is 2 (see Table 1).

**TABLE 1:** Two cases of mNGS and culture and drug sensitivity testing in pediatric patients.

	Case 1				Case 2			
	cultures		mNGS		cultures		mNGS	
CSF	KP(12/11/23) (12/19/23)		KP and SMA(12/15/23)		-		KP (4/10/24)	
Blood	-		—		-		—	
Sputum or Bulf	-		—		KP and Cs(4/09/24)		KP, Cs and Sp(4/09/24)	
Urine	—		—		CRKP		—	
	KP(Csf-lumbar puncture)		KP(Csf-drainage tube)		KP(Urine)		KP(Bulf)	
ATBIOTICS	sensitivity	Resistance	Sensitivity	Resistance	sensitivity	Resistance	sensitivity	Resistance
Ceftriaxone		√		√	√	√	√	√
Cefepime		√		√		√		√
Cefotetan		√		√		√		√
Cefoxitin		√		√		√		√
Amikacin		√		√		√		√
Levofloxacin		√		√		√		√
Meropenem		√		√		√		√
Tigecycline	√		√		—	—	√	
Polymyxin B		√		√	√		√	
CAZ/AVI	√					√		√
Types of CR	KPC+		KPC+		KPC+		KPC+	

NOTE:* SMA, stenotrophomonas maltophilia; Cs, Corynebacterium striatum; Sp, *Streptococcus* pneumonia,/not checked, - negative.

Based on clinical presentation, medical history and laboratory results, bacterial meningitis was confirmed. Empirical therapy with polymyxin B (40,000 U/kg/day q12h IV drip, 20,000 U intrathecal injection every day) was initiated. Unfortunately, the infant still experienced fever during the first 4 days of hospitalization, with temperature peaks gradually rising to 38.5°C.To further assess the bacterial resistance and virulence, metagenomic next-generation sequencing (mNGS) of CSF was performed detecting KP (807,646 reads, high confidence) and Stenotrophomonas maltophilia (498,170 reads, high confidence). The CRKP strain carried numerous resistance genes, including TEM-206, OXA-9, AAC (3)-IIc, tet(A), sul1, sul2, rmt B, Fos A3, TEM β-lactamase,Tamase, SHV β-lactamase and KPC β-lactamase as well as virulence factors Aerobactin and Sal, confirming a diagnosis of bacterial meningitis caused by CR-hvKP. After multidisciplinary discussions involving infectious disease specialists, clinical pharmacists, and neurologists, the patient was started on CAZ/AVI (187.5 mg/kg/day q8h IV drip) combined with compound sulfamethoxazole. After 72 h of treatment with CAZ/AVI and resettlement of drainage tube, the child’s temperature normalized. Follow-up CSF result showed improvement with pleocytosis ([WBC] 61/mm^3^, 80% polymorphonuclear cells), CSF glucose (2.26 mmol/L), and high CSF protein (503.5 mg/dL). The CSF culture still indicated KPC-KP which CAZ/AVI was sensitive. Therapy with CAZ/AVI was continued. Following an 18-day hospital stay, the child was cured after completing 14 days of CAZ/AVI and compound sulfanilamide therapy. CSF findings showed further improvement with pleocytosis (WBC 18/mm^3^, 2% polymorphonuclear cells), and CSF protein (0.9 mg/dL). The CSF culture was negative and repeat mNGS of the CSF did not detect KP or Stenotrophomonas maltophilia. The child was successfully discharged on the 23rd day after undergoing neurosurgical replacement of the external drainage tube. During postoperative follow-up, all repeat CSF cultures remained negative over the course of 1 month.

### Case 2

In April 2024, a 9-month-old female infant was admitted to the PICU due to recurrent high fever following laryngeal function reconstruction and laryngoplasty, and failure to be weaned off mechanical ventilation at the primary hospital (see Figure 1). Postoperatively, the infant developed a high fever and a urinary tract infection, with urine culture revealing CRKP (inhibition zone diameter was 20 mm) (see Table 1). Upon admission, invasive mechanical ventilation was continued, and the infant received ceftazidime (150 mg/kg/day q8h IV drip) combined with polymyxin B (40,000 U/kg/day q12h IV drip). A culture from the endotracheal tube tip later confirmed KPC-producing CRKP (inhibition zone diameter was 20 mm). On the third day of hospitalization, the infant developed recurrent seizures with a peak of 37.8°C, presenting with limb stiffness, tremors, fixed gaze, increased heart rate, and decreased oxygen saturation. CSF result showed pleocytosis with ([WBC] 140/mm^3^, 40% polymorphonuclear cells, CSF glucose 3.31 mmol/L, and CSF protein 0.54 mg/dL). Although CSF cultures were negative, mNGS detected KP (139 reads, moderate confidence). Given the positive CRKP cultures from both the urinary tract and endotracheal tube, the diagnosis of bacterial meningitis caused by CRKP was confirmed. Although no virulence genes were found on mNGS, the diagnosis was consistent with hypervirulent CRKP (hv-KP) meningitis, originating from a urinary tract infection. Despite initial treatment with polymyxin B, the infant continued to experience recurrent hyperthermia and convulsions. After multidisciplinary discussions involving infection specialists, clinical pharmacists, and neurologists, the antibiotic regimen was adjusted to CAZ/AVI (187.5 mg/kg/day every 8 h IV drip). After 48 h of CAZ/AVI therapy, the fever peaks subsided. Repeat CSF analysis showed 0 nucleated cells, with CSF protein at 0.62 mg/dL, glucose at 3.83 mmol/L, and chloride at 131.3 mmol/L. After 10 days of CAZ/AVI therapy, a follow-up CSF examination showed no nucleated cells, with CSF protein at 541.8 mg/L, glucose at 3.49 mmol/L, and chloride at 125.1 mmol/L. CSF culture was negative, and repeated mNGS did not detect KP. The infant did not develop fever and convulsions again and was discharged on day 29. A 3-month outpatient follow-up showed no adverse effects.

## Discussion

Meningitis caused by CRKP is associated with a high mortality rate, largely due to the increasing incidence of CR-hvKP strains (Huang et al., 2022). These infections progress rapidly and become severe. The patients have poor prognoses due to the bacteria’s high virulence and multidrug resistance. Current international and Chinese guidelines generally recommend the use of polymyxins or newer β-lactam/β-lactamase inhibitor combinations for treating intracranial infections caused by CRKP (Chinese Society of HematologyChinese Medical Association and Chinese Hematology AssociationChinese Medical Doctor Association, 2020; Tsuji et al., 2019). The choice of these drugs is based on their antibacterial spectrum and *in vitro* activity against CRKP. For example, polymyxin B is widely used due to its high activity against carbapenem-resistant organisms, although its neurotoxicity requires careful monitoring. Cefiderocol has been approved for Complicated Urinary Tract Infections (cUTI), Including Pyelonephritis in the United States, Europe, and Japan, but it has not yet been approved in China,and Its efficacy in CRKP meningitis is still uncertain. Aztreonam will be hydrolyzed by various enzymes other than metalloenzymes, so it is not a treatment option for CRKP that produces KPC. It is essential to consider the patient’s specific condition and consider multiple factors, including drug resistance, toxicity, clinical data, and drug availability, to develop a more scientific and effective treatment plan for intracranial infections caused by CRKP.

This study presents two pediatric cases of intracranial infections caused by CRKP. In one case, the infection was secondary to a urinary tract infection that spread hematogenously, while in the other, the CRKP-related intracranial infection following neurosurgery. *In vitro* culture and antimicrobial sensitivity testing revealed that in Case 1, the CRKP strain was resistant to both carbapenems and polymyxin B, but sensitive to CAZ/AVI. In Case 2, the strain was resistant to carbapenems and CAZ/AVI, but sensitive to polymyxin B. Although no virulence factors were detected in Case 2, the infection was considered CR-hvKP due to its hematogenous spread. Both children received polymyxin B for over 72 h, with one case also involving intrathecal and sheath injection administration. Both cases experienced recurrent fever and CSF cell counts did not decrease significantly. Currently, polymyxin is the primary drug utilized for intrathecal injection in the treatment of CRKP. However, it presents certain therapeutic hurdles including the potential for local irritation and the need for careful monitoring of drug levels to avoid toxicity. After consultation with a multidisciplinary team of critical care, neurology, and clinical pharmacy specialists, both patients were switched to CAZ/AVI. Within 14 days, significant improvements were observed in clinical symptoms, signs, and CSF analysis, with no long-term sequelae or drug-related adverse effects noted during follow-up. These findings suggest that CAZ/AVI may be a viable treatment option for pediatric intracranial infections caused by KPC-KP.

CAZ/AVI is a novel combination of β-lactam antibiotics and β-lactam inhibitors. AVI protects CAZ from hydrolysis by β-lactamase through inhibiting β-lactamase, thereby enhancing or restoring the antibacterial activity of CAZ (Ehmann et al., 2012; Zhanel et al., 2013). It is used to treat Gram-negative infections caused by KP, *Enterobacter cloacae*, *Escherichia coli*, *Proteus mirabilis* and *Pseudomonas aeruginosa* in adult patients with limited treatment options.

Our research group conducted a literature review using the keywords ‘intracranial infections caused by CRKP’ and “CAZ/AVI” and found several studies demonstrating the effective antibacterial activity of CAZ/AVI against CRKP (Xu et al., 2024; Zhang et al., 2024; Zhao et al., 2023; Yasmin et al., 2023; Pektezel et al., 2022; Zhou et al., 2021; Yasmin et al., 2020; Iosifidis et al., 2019; Holyk et al., 2018; Gofman et al., 2018). As shown in Table 2, most of the studies confirm that CAZ/AVI may be a preferred option for treating intracranial infection caused by CRKP. Zhao X. et al. showed that the clinical success rate was 76.2% (16/21), the microbial cure rate was 81.0% (17/21), and the all-cause mortality rate was 23.8% (5/21). The main phenotype of CRKP is KPC. The phenotypes of two studies are OXA and NDM (Pektezel et al., 2022; Zhao et al., 2023). However, most existing reports are limited to individual cases and primarily focus on adults following brain surgery, with few reports addressing its effectiveness in treating intracranial infection in children. In 2019, Iosifidis et al. (2019) reported a case of a 2.5-year-old female child who was admitted to PICU due to cerebral trauma. During treatment, the child developed a bloodstream infection and CNS infection caused by Pandrug-resistant KP. Initially treated with meropenem, ertapenem and, polymyxin B failed, as the clinical symptoms worsened. Antimicrobial sensitivity testing revealed resistant to multiple antibiotics, including cefepime, aztreonam, meropenem, imipenem, fosfomycin, cotrimoxazole, tigacycline and polymyxin B, only sensitivity to CAZ/AVI. Consequently, the child was treated with CAZ/AVI 700 mg q8h (based on her weight of 14 kg) and her symptoms improved within 2 days. Bacterial culture results were negative, and the child was discharged after 32 days of continuous CAZ/AVI treatment. However, 1 month later, the child was readmitted to the hospital due to the same drug-resistant bacterial infection. The patient was treated with a combination of CAZ/AVI, meropenem, and polymyxin B for 38 days, after which symptoms improved, the child was discharged. This case aligns with the treatment outcomes observed in the two pediatric patients in this study. Meanwhile, in this study, both patients were treated with CAZ/AVI alone and were cured without any long-term complications, indicating that CAZ/AVI is effective for intracranial infections caused by CRKP in children.

**TABLE 2:** Related researches on CAZ/AVI treatment of Central nervous system infection.

References	Population and case	Microbiological isolation	Enzyme/genotype	Antimicrobial susceptibility	Treatment for CAV/AVI	CAV/AVI duration	Outcome
Xu et al. (2024)	7 Adults	CRKP(4/7) CRPA(3/7)	—	Y(S)	CAV/AVI 1.25–2.5 g q8h (6cases received combination therapy based on CAV/AVI, and 1 case received alone)	The median duration 17days (8–22 days)	Cure
Zhang et al. (2024)	1 Adults	PDR-KP	—	Y(S)	CAV/AVI2.5g q8h + poly B 750,000 q12h + poly B 50,000 intrathecal qd	18 days	Cure
Zhao et al. (2023)	21 Adults	CRKP(21/21)	KPC(20/21) NDM(1/21)	Y(20/21) N(1/21)	18 cases received combination therapy based on CAV/AVI, and 3 cases received CAV/AVI treatment alone.	3–54 days (Median 26.6days)	Clinical success 76.2%(16/21) Microbiologic cure 81.0%(17/21) All-cause Mortality 23.8%(5/21)
Yasmin et al. (2023)	3 Adults	CRKP(1/3) DTR-PA(1/3) CRE(1/3)	KPC(2/3)	Y(S)	1.CAV/AVI2.5g q8h + amikacin intrathecal qd 2.CAV/AVI2.5 g q8h + CMS 4.5 MIU q12h + intrathecal CMS 125000 IU q12h 3.CAV/AVI2.5g q8h + CMS load 9 MIU sustain 4.5 MIU q12h + intrathecal CMS 125000 IU q12h	At least 3 weeks	Cure
(Pektezel et al., 2022)	1 Adults	CRKP	bla_OXA-48_	Y(S)	CAV/AVI + TMP-SMZ + amikacin intrathecal	30 days	Cure
Zhou et al. (2021)	3 Adults	XDP-KP(1/3) MDR-PA(1/3) MDR-PA&CRKP(1/3)	KPC	Y(S)	1.Meropene 2g + CAV/AVI 2.5 q8h 2.CAV/AVI2.5g q8h + amikacin 400 mg qd 3.CAV/AVI2.5g q8h + amikacin 600 mg qd	19–54 days	Cure
Yasmin et al. (2020)	1 Adults	MDR-KP	bla_KPC-3_	Y(S)	CAV/AVI2.5g q8h + amikacin intrathecal	10 days	Cure
Iosifidis et al. (2019)	1 Child*	CRKP	—	Y(S)	CAV/AVI62.5 mg/kg q8h	(Median) 14 days	Cure
Holyk et al. (2018)	1 Adults	CRKP	—	Y(S)	CAV/AVI + intrathecal gentamicin	21 days	Cure
Gofman et al. (2018)	1 Adults	CRKP&PA	—	Y(S)	CAV/AVI2.5g q8h + amikacin 30 mg intrathecal qd	6 weeks	Cure

NOTE:*, Only 1 in 8 cases was Central nervous system infection;/, not checked.

Although this study preliminarily demonstrated the potential efficacy of CAZ/AVI in treating CRKP-induced meningitis through cases report, several limitations remain that require further investigation. Firstly, the small number of case reports limits the generalizability and reliability of the findings. The rarity of meningitis, combined with the complexity of treating CRKP infections, makes it challenging to draw definitive conclusions from just a few cases. Secondly, this study lacks a control group, preventing direct comparisons of efficacy and safety between CAZ/AVI and other treatment regimens. Furthermore, this study has not yet explored the pharmacokinetics and pharmacodynamics of CAV/AVI, which are particularly important in the treatment of intracranial infections. Effective meningitis treatment should address not only short-term outcomes but also long-term prognosis and quality of life. The limited follow-up in these case reports makes it difficult to fully assess the long-term efficacy of CAZ/AVI in treating CRKP meningitis. In summary, although CAZ/AVI shows promise in treating CRKP meningitis, current research has significant limitations. Larger rigorously designed clinical trials are needed to further validate its efficacy and safety.

## Data Availability

The original contributions presented in the study are included in the article/supplementary material, further inquiries can be directed to the corresponding author.

## Abbreviations

CRKP, Carbapenem-resistant *Klebsiella* pneumonia; CNS, Central nervous system; CSF, Cerebrospinal fluid; HvKP, Hypervirulent *klebsiella* pneumonia; CR-hvKP, Hypervirulent carbapenem-resistant *klebsiella* pneumonia; CRGNB, Carbapenem-resistant Gram-negative bacteria; CAZ/AVI, Ceftazidime/avibactam; WBC, White blood cell count; mNGS, Metagenomic next-generation sequencing.
